# Advances in the study of exosomes derived from mesenchymal stem cells and cardiac cells for the treatment of myocardial infarction

**DOI:** 10.1186/s12964-023-01227-9

**Published:** 2023-08-14

**Authors:** Yuchang Liu, Minrui Wang, Yang Yu, Chunhong Li, Chunxiang Zhang

**Affiliations:** 1 Department of Pharmaceutical Sciences, School of Pharmacy, Southwest Medical University, Luzhou, 646000 Sichuan China; 2 School of Basic Medical Science, Southwest Medical University, Luzhou, 646000 Sichuan China; 3 Department of Cardiology, The Affiliated Hospital of Southwest Medical University, Luzhou, 646000 Sichuan China; 4 The Key Laboratory of Medical Electrophysiology of the Ministry of Education, Southwest Medical University, Luzhou, 646000 Sichuan China; 5 Laboratory of Nucleic Acids in Medicine for National High-Level Talents, Southwest Medical University, Luzhou, 646000 Sichuan China

**Keywords:** Exosomes, Myocardial infarction, Mesenchymal stem cells, Cardiac cells

## Abstract

Acute myocardial infarction has long been the leading cause of death in coronary heart disease, which is characterized by irreversible cardiomyocyte death and restricted blood supply. Conventional reperfusion therapy can further aggravate myocardial injury. Stem cell therapy, especially with mesenchymal stem cells (MSCs), has emerged as a promising approach to promote cardiac repair and improve cardiac function. MSCs may induce these effects by secreting exosomes containing therapeutically active RNA, proteins and lipids. Notably, normal cardiac function depends on intracardiac paracrine signaling via exosomes, and exosomes secreted by cardiac cells can partially reflect changes in the heart during disease, so analyzing these vesicles may provide valuable insights into the pathology of myocardial infarction as well as guide the development of new treatments. The present review examines how exosomes produced by MSCs and cardiac cells may influence injury after myocardial infarction and serve as therapies against such injury.

**Graphical Abstract:**

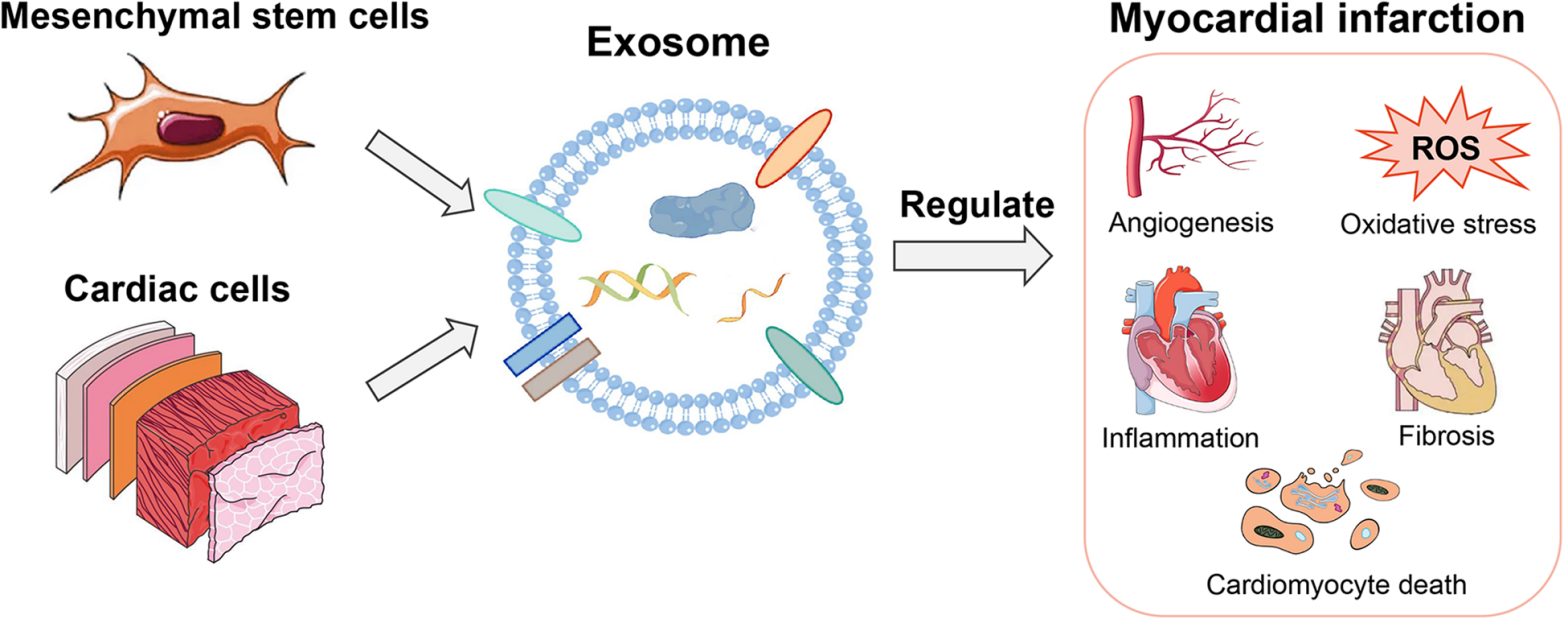

Video Abstract

**Supplementary Information:**

The online version contains supplementary material available at 10.1186/s12964-023-01227-9.

## Introduction

Despite considerable improvements in healthcare worldwide, acute myocardial infarction (AMI), defined as the death of myocardial cells due to prolonged ischemia, is the most serious manifestation of coronary artery disease and the primary cause of associated death [1, 2]. Cells of adult heart cannot regenerate after loss due to ischemia, so myocardial infarction (MI) can cause the irreversible loss of too many cardiomyocytes, leading to left ventricular remodeling and even progressive heart failure [3].

The current standard treatment for AMI is reperfusion, but this can aggravate cardiomyocyte dysfunction and tissue injury, a process known as ischemia/reperfusion (I/R) injury [4]. Such injury can lead initially to oxidative stress and inflammation, and can rapidly develop into apoptosis and necrosis [5]. This highlights the need to develop new treatments to reduce cardiomyocyte death and promote cardiac repair after MI.

A fruitful approach may be to exploit exosomes, extracellular vesicles containing regulatory RNAs, lipids and proteins that are normally secreted by many types of cells, including cardiomyocytes, endothelial cells (ECs), immune cells, and stem cells. Exosomes serve as important paracrine signaling vehicles, helping to promote angiogenesis, inhibit harmful ventricular remodeling, improve heart function, inhibit local inflammation and regulate immune responses [6].

This review provides an overview of recent developments in our understanding of exosomes secreted by mesenchymal stem cells (MSCs) and cardiac cells over the past five years. Certain exosomes exhibit great potential in the treatment of MI, while others impact the progression and prognosis of MI [6, 7]. Although no studies have compared or independently described exosomes of animal and human origin, multiple studies have demonstrated that exosomes derived from both human and rat MSCs attenuate cardiomyocyte apoptosis in rats with MI [8, 9]. Consequently, we postulate that exosomes of homologous cellular origin from different races may share similar biological properties. Furthermore, this review examines the prospects and challenges of utilizing exosomes to treat patients after MI, with the aim of providing valuable information for future research that compares the specific biological functions of exosomes of animal and human origin.

## Characteristics of exosomes 

Exosomes are a type of extracellular vesicles (EVs) with diameters between 40 and 160 nm [10]. They are secreted by dendritic cells, mast cells, platelets, and MSCs as well as other cell types, and they are found in most body fluids of humans and animals, including plasma, serum, saliva, amniotic fluid, breast milk, and urine [11]. They were discovered by Pan and Johnstone during their studies of how sheep reticulocytes mature into erythrocytes [12]. Since its discovery, exosomes have been extensively studied for their biological characteristics, function, and potential clinical application.

Exosomes are generated from multivesicular endosomes that fuse with the cytoplasmic membrane and are secreted, instead of being degraded by lysosomes or autophagosomes [13]. Exosomes can enter recipient cells by employing different mechanisms such as endocytosis, direct fusion with membranes, or binding to receptors on the cell surface, and thus achieve their complex biological effects (Table 1) [14–18]. Exosomal cargo comprises cell surface and intracellular proteins, RNA, DNA, lipids, and metabolites (e.g. amino acids, ATP, amides), and its type and content are influenced by donor cells, microenvironment, or physiological conditions. By transferring these biomolecules containing important information to different cells, exosomes are able to influence gene transcription and cell proliferation [10]. Investigation of exosomal cargo and its functions may provide insight into many cell–cell communication processes in health and disease.

**Table 1:** The main mechanism of exosome entering the recipient cell

Mechanism		General mediators	Characteristics
Soluble and juxtacrine signalling		TNF, Fasl	Exosomes that bind to recipient cells elicit signal transduction through intracellular signaling pathways and are released
Fusion		Tetraspanins	Exosomes fuse with the plasma membrane of the cell
Endocytosis	Receptor-mediated endocytosis	Lipid rafts, clathrin, caveolin	This category of endocytosis is common and selective, focusing on specific cellular proteins that facilitate the uptake of particles
	Phagocytosis	Actin, complement receptors	Phagocytosis is typically performed by phagocytic cells, such as macrophages, which non-specifically engulf extracellular material, resulting in exosome uptake
	Macropinocytosis	Actin, Na^+^, PI3K	Macropinocytosis is similar to phagocytosis but does not require direct contact with the internalized material

*Fasl* Factor related apoptosis ligand, *PI3K* Phosphatidylinositol3-kinase, *TNF* tumor necrosis factor

Biomarkers of exosomes are also a key research focus. The complexity of exosomes is further amplified by their specific biomarkers, which possess characteristic compositions of donor cells. For example, tumor-secreted exosomes are usually highly expressed in heat shock proteins, whereas B cell-secreted exosomes contain large amounts of tetraspanins [19, 20]. Nevertheless, the biomarkers of exosomes also exhibit homogeneity. Typical biomarkers of exosomes include proteins such as CD9, CD81, CD63, ceramide, tumor susceptibility gene 101 (TSG101), as well as apoptosis-linked gene 2-interacting protein X (ALIX), all of which are involved in the origin and biogenesis of exosomes (Table 2) [14, 21–23]. The properties of the biomarkers suggest an important role in the identification and purification of exosomes, and also give exosomes great potential for clinical applications such as disease diagnosis and targeted therapies.

**Table 2:** Typical biomarkers of exosome

Name	Classification	Function
CD63	Tetraspanin	Cell targeting and adhesion
CD9		
CD81		
TSG101	ESCRT protein	Origin and biogenesis of exosome
ALIX	ESCRT-associated protein	
Flotillin	Membrane protein	Membrane transport/fusion
HSP70	Heat shock protein	Molecular chaperone
HSP90		

*ALIX* Apoptosis-linked gene 2-interacting protein X, *ESCRT* Endosomal sorting complexes required for transport, *HSP70* heat shock protein 70, *HSP90* heat shock protein 90, *TSG101* tumor susceptibility gene 101

Exosomes possess a wide and unique advantage in drug delivery and treatment due to their endogeneity and heterogeneity in applied research. Their endogeneity provides superior biocompatibility and low toxicity compared to common nanocarriers, making them excellent gene drug candidate vectors [24]. On the other hand, heterogeneity is one of the most important features of exosomes, which largely determines their function. The heterogeneity of exosomes is reflected by various factors, such as multiple mechanisms of exosome biogenesis, diverse cellular sources, content, and functional heterogeneity in their impact on recipient cells [25]. A deeper understanding of exosome characteristics will significantly advance related research in this field.

## Exosomes derived from MSCs

**Fig. 1:**
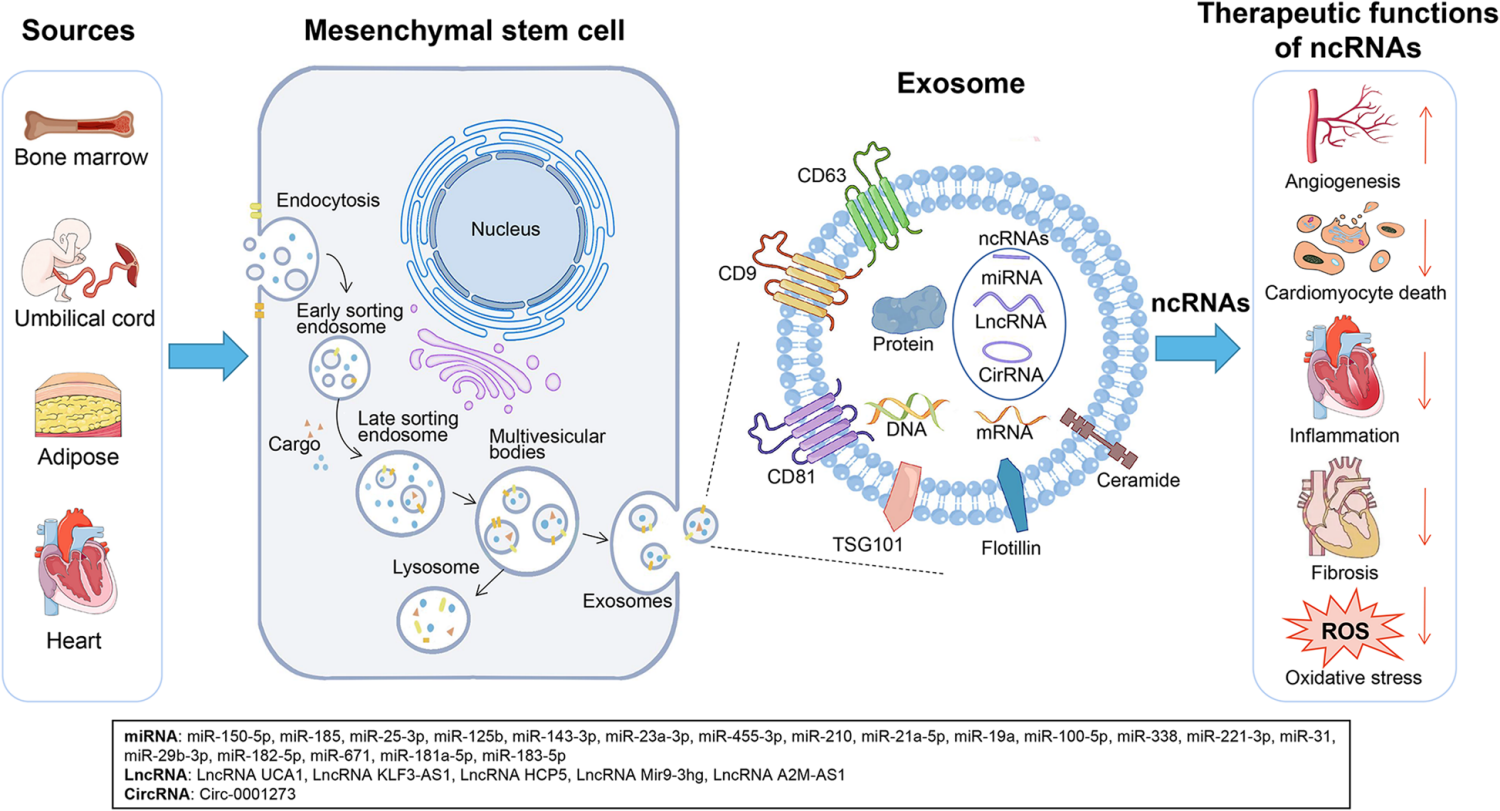
Biogenesis of mesenchymal stem cell-derived exosomes and potentially therapeutic functions of non-coding RNAs they carry. The process of exosome biogenesis involves a double invagination of the plasma membrane and the formation of intracellular multivesicular bodies (MVBs). The plasma membrane invaginates for the first time and undergoes endocytosis, generating early sorting endosomes in the cytoplasm. Early sorting endosomes can mature into late sorting endosomes, which further generate MVBs. MVBs are formed by the second invagination of the plasma membrane which can either fuse with lysosomes or autophagosomes to be degraded or fuse with the plasma membrane to release intraluminal vesicles (i.e., exosomes). CircRNA, circular RNA; LncRNA, long non-coding RNA; miRNA, microRNA; ncRNA, non-coding RNA; ROS, reactive oxygen species, TSG101, Tumor susceptibility gene 101

If the ability to divide and differentiate into multiple cell types makes stem cells particularly interesting for medicine [26], then exosomes that can promote the repair and regeneration of injured tissue are also particularly attractive. Among the various types of stem cells that have shown therapeutic potential [27]. MSCs stand out because they have the potential of multi-directional differentiation and can function in a variety of adult tissues, including bone marrow, fat, umbilical cord blood and placenta [28–31]. Their ability to promote angiogenesis and recovery of ischemic tissue make them attractive for treating heart disease [32]. They secrete exosomes containing a broad array of regulatory RNAs with potential to ameliorate tissue injury after MI [33]. Exosomes from different MSCs mostly have common therapeutic effects, but their specific mechanisms are slightly different, which is closely related to their contents [34–37]. Multiple studies have shown that functionally diverse non-coding RNAs (ncRNAs) in exosomal cargoes can account for the vast majority of their therapeutic effects, and the mechanisms responsible for their effects have been explored in depth. Figure 1 depicts the biogenesis of exosome, and the potentially therapeutic functions of ncRNAs in exosomes derived from MSCs. We aim to elucidate the molecular mechanisms underlying the action of MSC-derived exosomes in order to facilitate their clinical translation.

## Exosomes derived from bone marrow MSCs

Bone marrow MSCs (BMSCs) show weak immunogenicity, multidirectional differentiation and high transplantability, making them well-suited for treating cardiovascular diseases. BMSCs have been shown to be safe in clinical studies [38], and exosomes derived from BMSCs can influence various pathways in cardiomyocytes and macrophages to reduce myocardial injury and improve cardiac function. On the whole, exosomes derived from BMSCs can achieve good therapeutic effects through the joint action of various mechanisms.

## Inhibition of cardiomyocyte death

Reperfusion of ischemic myocardium can exacerbate apoptosis, necrosis and cell death [39]. MicroRNA (miRNA) is a small (~ 21 nucleotide) ncRNA, and miRNAs in BMSC-derived exosomes can coordinately inhibit genes that drive cardiomyocyte apoptosis and heart injury. For example, miR-150-5p in BMSC-derived exosomes downregulates the pro-apoptotic B-cell lymphoma-associated X (Bax) protein to reduce cardiomyocyte apoptosis and improve cardiac function in a mouse model of MI [40]. BMSC-derived exosomes also carry miR-183-5p, which inhibits expression of forkhead box O1 (FOXO1) to reduce apoptosis and oxidative stress in cardiomyocytes [41]; miR-21a-5p, which downregulates the pro-apoptotic genes programmed cell death 4 (*Pdcd4*) and factor related apoptosis ligand (*Fasl*), protecting infarcted myocardium [42]; miR-25-3p, which downregulates the pro-apoptotic genes *Fasl* and phosphatase and tensin homolog (*Pten*) [43]; and miR-125b, which downregulates the pro-apoptotic genes *P53* and B-cell lymphoma-2 antagonist/killer 1 (*Bak1*) [44]. This reflects the complexity of the signaling network in target cells regulated by exosomes. It involves not only multiple miRNAs acting on the same gene or pathway, but also the generation of synergistic effects through a single miRNA targeting multiple gene products.

During myocardial I/R injury, the activation of NOD-like receptor thermal protein domain associated protein 3 (NLRP3) inflammasomes can lead to the secretion of inflammatory cytokines and pyroptosis of cardiomyocytes [45]. BMSC-derived exosomes containing miR-182-5p can inhibit the expression of pro-pyroptosis protein gasdermin D (GSDMD) and toll-like receptor 4 (TLR4), as well as the TLR4/nuclear factor-κB (NF-κB) signaling pathway, thereby exerting powerful anti-inflammatory and anti-pyroptosis effects [46, 47].

**Fig. 2:**
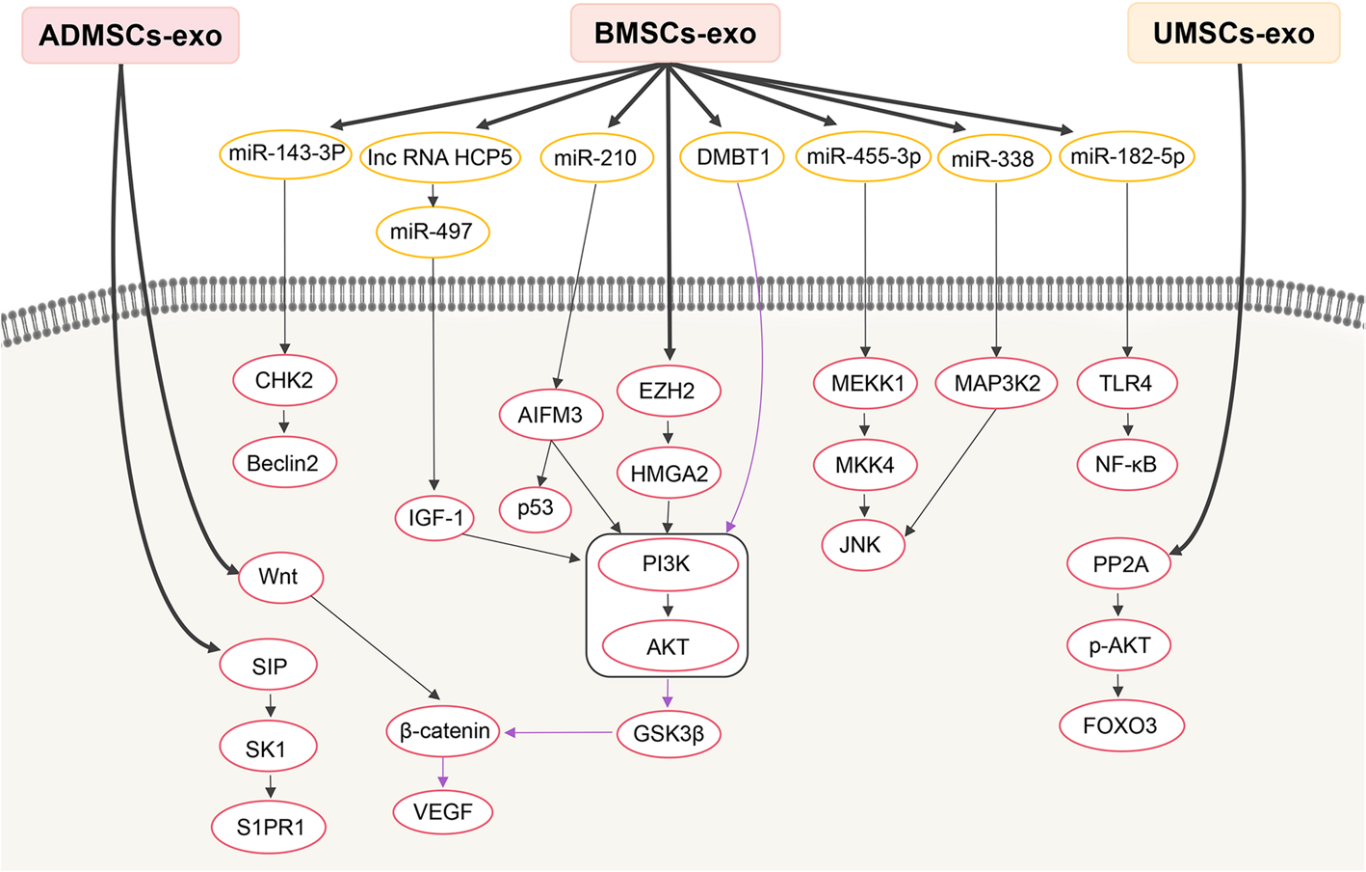
Selected signaling pathways through which exosomes from MSCs exert therapeutic effects. ADMSC-exo, exosomes derived from adipose mesenchymal stem cells; AIFM3, apoptosis-inducing factor, mitochondrion-associated 3; AKT, protein kinase B; BMSC-exo, exosomes derived from bone marrow mesenchymal stem cells; CHK2, checkpoint kinase 2; DMBT1, deleted in malignant brain tumors 1; EZH2, enhancer of zeste 2 polycomb repressive complex 2; FOXO3, forkhead box O3; GSK3β, glycogen synthase kinase 3β; HMGA2, high mobility group AT-hook 2; IGF-1, insulin-like growth factor-1; JNK, C-Jun N-terminal kinase; MAP3K2, mitogen-activated protein kinase kinase kinase 2; MEKK1, mitogen-activated protein kinase kinase kinase 1; MKK4, mitogen-activated protein kinase kinase 4; NF-κB, nuclear factor-κB; p-AKT, phosphorylated protein kinase B; PI3K, phosphatidylinositol3-kinase; PP2A, protein phospholipase 2A; S1P, sphingosine 1-phosphate; S1PR1, sphingosine-1-phosphate receptor 1; SK1, sphingosine kinase 1; TLR4, toll-like receptor 4; UMSC-exo, exosomes derived from umbilical cord mesenchymal stem cells

Interestingly, BMSC-derived exosomes contain miRNAs that regulate several therapeutically useful signaling pathways (Fig. 2). For instance, regulatory RNAs in BMSC-derived exosomes can influence the phosphatidylinositol3-kinase/ protein kinase B (PI3K/AKT) pathway to attenuate cardiac fibrosis after MI and myocardial injury after hypoxia [48, 49]. Additionally, several miRNAs can regulate the C-Jun N-terminal kinase (JNK) pathway, which is a key regulatory pathway of apoptosis. For example, miR-338 acts through the mitogen-activated protein kinase 2/JNK (MAP3K2/JNK) signaling pathway to attenuate cardiomyocyte apoptosis, while miR-455-3p can inhibits mitogen-activated protein kinase 1/mitogen-activated protein kinase 4/JNK (MEKK1-MKK4-JNK) signaling pathway [50, 51]. Moreover, miR-143-3p regulates autophagy by inhibiting the checkpoint kinase 2 (CHK2)/Beclin2 pathway to effectively inhibit cardiomyocyte apoptosis [52].

BMSC-derived exosomes also carry long non-coding RNAs (lncRNAs), which usually regulate gene expression by interacting with miRNAs. For example, lncRNA KLF3-AS1 binds to miR-138-5p to upregulate sirtuin 1 (Sirt1), thereby inhibiting pyroptosis and slowing progression of injury following MI [53]. Another study is lncA2M-AS1, which sponges miR-556-5p to upregulate the anti-apoptotic protein X-linked inhibitor of apoptosis protein (XIAP), reducing myocardial apoptosis and oxidative stress caused by I/R injury [54]. By targeting miR-497/IGF1 axis, the lncRNA HCP5P in BMSC-derived exosomes also inhibits the PI3K/AKT signaling pathway, thereby increasing the viability of cardiomyocytes following hypoxia/reperfusion and decreasing apoptosis [55].

MI kills cardiomyocytes not only by promoting autophagy and apoptosis but also ferroptosis, in which the intracellular glutathione-dependent antioxidant defense system is defective, resulting in the accumulation of toxic reactive oxygen species (ROS) and iron [56]. BMSC-derived exosomes contain lncRNA Mir9-3hg that upregulates peroxoredoxin 6 (PRDX6) to decrease levels of iron and ROS in cardiomyocytes after I/R [57]. These endogenous lncRNAs contained within exosomes serve as natural therapeutic agents and offer a highly valuable gene therapy strategy for the treatment of cardiovascular diseases.

## Promotion of angiogenesis

MI injures the vascular endothelium and restricts myocardial blood supply, so rescuing angiogenesis is critical [58]. BMSC-derived exosomes upregulate platelet-derived growth factor receptor-β (PDGFR-β) to enhance microvascular regeneration, inhibit fibrosis, and maintain long-term cardiac function after I/R injury [59]. This study also found that BMSC-exo had even better therapeutic effects than BMSCs themselves, suggesting that exosomes have great potential for future practical applications. These effects appear to depend on miR-221-3p, since lower levels of this miRNA in exosomes lead to weaker therapeutic effects [60].

While naturally occurring exosomes show therapeutic potential, genetic engineering can be used to create more potent exosomes in the laboratory. For example, infecting BMSCs with recombinant lentivirus allowed researchers to create exosomes containing abundant hypoxia-inducible factor 1 (HIF-1α), which rescued angiogenic, migratory and proliferative functions of hypoxia-injured human umbilical vein endothelial cells (HUVECs), and led to neovessel formation and inhibition of fibrosis in a rat model of MI [58]. In another approach, exposing BMSCs to ischemic heart extract rich in interleukin (IL)-22 led to exosomes carrying high levels of deleted in malignant brain tumors 1 (DMBT1), which can activate PI3K-AKT/ glycogen synthase kinase 3β(GSK3β)/β-catenin/ vascular endothlial growth factor (VEGF) signaling to enhance migration and proliferation of HUVECs [34].

## Inhibition of inflammation

Death of cardiomyocytes during myocardial I/R injury activates an inflammatory response that has a significant impact on the final extent of myocardial injury. Activation of inflammasomes can induce pyroptosis, leading to the release of intracellular contents into the extracellular environment. This, in turn, activates the inflammatory response and further exacerbates myocardial injury [53, 61]. Excessive or prolonged inflammation degrades the extracellular matrix, leading to ventricular remodeling [62]. Therefore, treating MI requires timely suppression of the inflammatory response.

Macrophages play a critical role in the progression and regression of inflammation [63]. Macrophages can be broadly divided into two types: M1 or “classically activated” macrophages secrete large amounts of pro-inflammatory factors, while M2 or “alternatively activated” macrophages produce various anti-inflammatory factors and growth factors. Soon after MI, M1 macrophages are recruited into the infarcted myocardium and exhibit strong phagocytic activity and pro-inflammatory activity, after which M2 macrophages come to dominate and resolve inflammation to allow myocardial repair [64]. BMSC-derived exosomes have been shown to activate AKT1/AKT2 signaling and the nuclear factor erythroid 2-related factor 2/heme oxygenase 1 (NRF2/HO-1) axis, while inhibiting NF-κB signaling. This shift in signaling pathways can change macrophages from an M1 to an M2 phenotype, which helps to reduce inflammation after MI [65, 66]. Treating BMSCs with fibronectin type III domain-containing protein 5 (FNDC5) can further enhance the ability of exosomes to promote this shift in macrophage phenotype [66].

BMSC-derived exosomes can also reduce the activity of the NLRP3 inflammasome in a mouse model of left coronary artery ligation [67]. As well, they carry miR-181a-5p that downregulates activating transcription factor 2 (ATF2) to protect the myocardium [68]. Exosomes carrying higher levels of this miRNA can be prepared by first treating the BMSCs with lipopolysaccharide [68]. Future research should continue to explore how to improve the therapeutic effects of exosomes by pretreating the parental cells. For example, studies should explore pretreating MSCs with inflammatory factors that mimic the inflammatory environment after MI, or with stromal factors or other cytokines that promote MSC growth.

## Other effects

BMSC-derived exosomes can inhibit harmful ventricular remodeling. They contain miR-185, which downregulates suppressor of cytokine signaling 2 (SOCS2), an inhibitor of growth hormone/IGF signaling [69]; and miR-29b-3p, which downregulates A disintegrin and metalloproteinase with thrombospondin 16 (ADAMTS16), blocking myocardial fibrosis and promoting angiogenesis in a rat model of MI [70].

BMSCs have been shown to promote myocardial recovery after infarction by influencing the renin-angiotensin system (RAS). Following MI, the activation of the neuro-humoral regulation mechanism, particularly the RAS, can accelerate the process of ventricular remodeling [71], which is associated with poor prognosis in MI patients [72]. Angiotensin-converting-enzyme inhibitors and angiotensin-receptor blockers, which can inhibit the RAS, occupy an important part in the pharmacotherapy of MI [73]. BMSC-derived exosomes accelerate the conversion of angiotensin II to angiotensin-(1-7), reducing the adverse effects of angiotensin II on cardiomyocytes and improving cardiac remodeling, as well as protecting cardiomyocytes from oxidative stress and associated apoptosis [72, 74]. This humoral therapeutic mechanism warrants further exploration in future research.

Work to explore the therapeutic potential of BMSC-derived exosomes should also explore their combination with BSMCs themselves. One study found that first injecting the exosomes followed by the cells effectively reduced scar size and restored cardiac function after acute MI [75]. Those authors proposed that the exosomes dampened inflammation in the heart and thereby created an environment to ensure survival of the subsequently injected BMSCs. This study not only demonstrates the effectiveness of exosome therapy, but also provides additional possibilities for future methods of clinical application of exosomes.

## Exosomes derived from adipose MSCs

Adipose MSCs (ADMSCs) have similar biological properties and multidirectional differentiation potential as BMSCs [76]. The source tissue is abundant and exosomes can be extracted from it efficiently and painlessly [77]. For these reasons, ADMSCs may replace BMSCs as a new stem cell source for regenerative medicine.

## Inhibition of cardiomyocyte apoptosis

Exosomes derived from ADMSCs contain miR-671, which downregulates transforming growth factor β receptor 2 (TGFBR2) and decreases mothers against decapentaplegic homolog (Smad2) phosphorylation, thereby reducing cardiomyocyte apoptosis, fibrosis and inflammation [78]. They also attenuate apoptosis after I/R and protect ischemic myocardium by activating the Wnt/β-catenin signaling pathway [35]. It is important to note that the Wnt/β-catenin signaling pathway influences macrophage polarization, angiogenesis, and other aspects [79, 80]. However, this study only examines its effects from one perspective, and a comprehensive and in-depth investigation could provide a fuller understanding of its role.

## Inhibition of inflammation

Similar to BMSC exosomes, exosomes derived from ADMSCs can shift macrophages toward an M2 phenotype and inhibit fibrosis and inflammation after MI, but the specific mechanisms may be different. Deng et al. found that this effect is achieved by activating the sphingosine 1-phosphate/sphingosine kinase 1/sphingosine-1-phosphate receptor 1 (S1P/SK1/S1PR1) axis [81].

## Promotion of angiogenesis

In hypoxic/ischemic environment, HIF-1 can drive the transactivation of dozens of genes involved in angiogenesis and play a critical role in mediating cardioprotection [82, 83]. Exosomes derived from ADMSCs contain miR-31, which downregulates an asparagine hydroxylase that blocks the binding of HIF-1α to coactivator p300, thereby “rescuing” expression of angiogenic genes [83].

## Exosomes derived from umbilical cord MSCs

Several studies indicate that the benefits of MSCs are due mainly to paracrine mediators contained within vesicles that they secrete [84]. Umbilical cord MSCs (UMSCs) stronger paracrine action and may induce even stronger therapeutic effects than MSCs from bone marrow and adipose [85].

## Inhibition of myocardial cell death

Exosomes derived from UMSCs exhibit a potential to promote cardiomyocyte survival after MI by inhibiting various cell death pathways such as apoptosis, autophagy, pyroptosis, and ferroptosis. They contain both miR-19a and circular RNA circ-0001273 that attenuate myocardial injury and inhibit cardiomyocyte apoptosis, which act by downregulating the transcription factor SRY (sex determining region Y)-box 6 (SOX6) and inhibiting miR-199b-3p, respectively [86, 87]. Moreover, UMSC-derived exosomes can promote the expression of Smad7 by inhibiting miR-125b-5p in the infarcted myocardium, providing additional myocardial protection [9]. They also carry lncRNA UCA1, which inhibits both autophagy and apoptosis [88]. Interestingly, Sun et al. showed that lncRNA UCA1 in exosomes secreted by hypoxic MSCs also achieved myocardial protection by sponging miR-873-5p [89]. Although this study does not specify the origin of MSCs, it suggests the importance of exploring the functional diversity of ncRNAs in exosomes.

Furthermore, UMSC-derived exosomes contain miR-100-5p that downregulates forkhead box O3 (FOXO3), which suppresses inflammation by suppressing NLRP3 inflammasome activation and protects cardiomyocytes from pyroptosis and injury [90]. By delivering miR-23a-3p, the exosomes can also inhibit ferroptosis by targeting the divalent metal transporter recombinant divalent metal transporter 1 (DMT1), which promotes iron accumulation in cardiomyocytes [91].

## Inhibition of inflammation

In contrast to regulation of macrophage polarization, exosomes derived from UMSCs exert anti-inflammatory effects through cardiac fibroblasts (CFs). Activated CFs after MI are characterized by fibroblast-myofibroblast transdifferentiation, accelerated proliferation, and extracellular matrix accumulation, which are essential for the dynamic homeostasis and remodeling of cardiac tissue, cardiomyocyte proliferation and angiogenesis [92, 93]. Analogous to macrophages, these fibroblasts adopt a pro-inflammatory phenotype soon after MI, after which they differentiate into myofibroblasts, which secrete anti-inflammatory factors and extracellular matrix proteins to repair and stabilize cardiac tissue [94]. Exosomes from human UMSCs promote differentiation of CFs toward myofibroblasts, thus attenuating the inflammatory response, reducing cardiomyocyte apoptosis, and promoting cardiac repair [95].

These exosomes may also inhibit inflammation and promote repair by activating regulatory T cells through a mechanism involving activation of protein phospholipase 2A (PP2A)/p-AKT/FOXO3 signaling [96]. That study demonstrated not only the potential for exosome-mediated immunomodulation but also the effectiveness of intrapericardial injection of exosomes.

## Other effects

Exosomes derived from UMSCs contain tissue inhibitor of metalloproteinase 2 (TIMP2), which inhibits matrix metalloproteinases and thereby inhibits harmful remodeling of the extracellular matrix and deposition of collagen, and the exosomes promote ECs proliferation and migration in damaged myocardium, leading to angiogenesis, while also inhibiting apoptosis [97, 98]. That study showed that levels of TIMP2 in exosomes can be increased by overexpressing TIMP2 in the parental UMSCs.

Notably, exosomes derived from UMSCs can also repair myocardium by affecting the function of other MSCs. Exosomes from UMSCs can enhance the ability of BMSCs to repair myocardium by delivering miR-136, which downregulates apoptotic protease activating factor- 1 (Apaf1) [99].

## Exosomes derived from cardiac MSCs

Cardiac MSCs play an important role in various aspects of ventricular remodeling, angiogenesis, and myocardial repair after MI [100–102]. Cardiac MSCs maintain cardiac homeostasis and promote cardiac repair through paracrine signaling [103]. Exosomes from cardiac MSCs can inhibit apoptosis, enhance myocardial angiogenesis and increase proliferation of cardiomyocytes in ischemic myocardium [104]. However, the related studies of cardiac MSC-derived exosomes are still limited, and further exploration is needed.

## Exosomes derived from cardiac cells

The ischemic-hypoxic microenvironment during MI induces apoptosis of most cell types within the heart, including cardiomyocytes, ECs, and macrophages [105]. Apoptosis of cardiomyocytes induces CFs to proliferate, leading to myocardial remodeling and impairing cardiac function. Exosomes produced by cardiac cells, especially cardiomyocytes, are an important source of exosomes in the heart and have a regulatory effect on cardiac function. For example, cardiomyocytes and ECs in the heart produce abundant exosomes under normal conditions and after MI [106]; and exosomes produced by CFs regulate cardiac hypertrophy [107]. Understanding the range of exosomes produced by cardiac cells and their molecular contents is important for exploiting them as potential therapies following MI (Table 3).

**Table 3:** Sources, cargo, pathways and biological effects of exosomes secreted by cardiac cells under the indicated conditions

Source of exosomes	Condition	Cargos	Pathways	Biological effects	Reference
Cardiomyocytes	MI	miR-328-3p↑	miR-328-3p/Caspase	apoptosis↑	[108]
Cardiomyocytes	MI	miR-19a-3p↑	miR-19a-3p/HIF-1α	angiogenesis↓	[109]
Cardiomyocytes	Hypoxia	lncRNA AK139128↑	not investigated	apoptosis↑	[110]
Cardiomyocytes	Ferroptosis	miR-106b-3p↓	miR-106b-3p/Wnt/macrophage polarization to M1	inflammation↑	[80]
Cardiomyocytes	Hypoxia	not investigated	macrophage polarization to M2	inflammation↓	[111]
Cardiomyocytes	Hypoxia	miR-208a	miR-208a/Dyrk2	fibrosis↑	[112]
Cardiomyocytes	Ischemia	miR-222; miR-143	not investigated	angiogenesis↑	[113]
Cardiomyocytes	Hypoxia	circHIPK3	circHIPK3/miR-29a/VEGFA	angiogenesis↑	[114]
Cardiomyocytes	Ischemia	lncRNA KLF3-AS1↑	lncRNA KLF3-AS1/miR-23c/STAT5B	apoptosis↓	[115]
CDCs	Normal/Hypoxia	Not investigated	not investigated	apoptosis↓	[116]
CDCs	Normal/Hypoxia	miR-126; miR-130a; miR-210	not investigated	angiogenesis↑	[117]
CDCs (EVs)	I/R	Y RNA fragment	Y RNA fragment/IL10	cardioprotection↑, infarct size↓	[118]
CDCs	Normal	miR-181b	miR-181b/PKCδ/macrophages polarization	cardioprotection↑, infarct size↓	[119]
ECs	MI	PFN2	PFN2/PI3K-PFN2-ERK	angiogenesis↑	[120]
ECs	Normal	not investigated	PI3K-AKT	apoptosis↓	[121]
ECs	Normal	LINC00174	LINC00174/SRSF1/P53/AKT-AMPK	apoptosis↓, autophagy↓	[122]
ECs (EVs)	MI	VCAM-1	VCAM-1/neutrophil mobilization	not investigated	[123]
Endothelial progenitor cells	Normal	miR-1246; miR-1290	miR-1246/ELF5, miR-1290/SP1	angiogenesis↑	[124]
Endothelial progenitor cells	Normal	miR-218-5p; miR-363–3	miR-218-5p/p53, miR-363–3/JMY	angiogenesis↑	[125]
Cardiac-resident progenitor cells	Patients undergoing heart surgery for aortic valve disease and/or coronary artery disease	PAPP-A	PAPP-A/IGF-1	apoptosis↓	[126]
Cardiac progenitor cells	Patients undergoing open-chest surgery	not investigated	MAPK/ERK1/2	inflammation↓, angiogenesis↑	[127]
Cardiac telocytes	Normal	miR-21-5p	miR-21-5p/Cdip1/caspase-3	apoptosis↓, angiogenesis↑	[128]
CFs	Hypoxia/reoxygenation	miR-133a↑	miR-133a/ELAVL1	pyroptosis↓	[129]
Epicardial cells	I/R	miR-30a; miR-100; miR-27a; miR-30e	not investigated	cardiomyocyte proliferation↑	[130]

*AKT* protein kinase B, *AMPK* adenosine 5 ‘-monophosphate (AMP)-activated protein kinase, *CDCs* cardiosphere-derived cells, *Cdip1* cell death inducing protein 1, *CFs* cardiac fibroblasts, *Dyrk2* dual specificity tyrosine (Y) phosphorylation regulated kinase 2, *ECs* endothelial cells, *ELAVL1* ELAV-like RNA-binding protein 1, *ELF5* E74-like factor 5, *ERK1/2* Extracellular regulated protein kinase 1/2, *EVs* extracellular vesicles, *HIF-1α* hypoxia-inducible factor 1, *I/R* ischemia/reperfusion, *IGF-1* insulin-like growth factor 1, *IL10* interleukin 10, *JMY* regulatory protein, *MAPK* mitogen-activated protein kinase, *MI* myocardial infarction, *PAPP-A* pregnancy-associated plasma protein-A, *PI3K* phosphatidylinositol3-kinase, *PKCδ* protein kinase C δ, *SP1* specificity protein 1, *SRSF1* serine and arginine rich splicing factor 1, *STAT5B* signal transducer and activator of transcription 5B, *PFN2* profilin 2, *VCAM-1* vascular cell adhesion molecule 1, *VEGFA* vascular endothlial growth factor A

## Exosomes derived from cardiomyocytes

Although cardiomyocytes are not typical secretory cells, they secrete exosomes that mediate communication between healthy and damaged cells under healthy and ischemic conditions [131]. These exosomes can also regulate cell proliferation, migration, differentiation, survival, and angiogenesis.

## Promotion of cell death

Exosomes from cardiomyocytes can exert both harmful and beneficial effects. For example, their content of miR-328-3p increases after infarction, and this miRNA activates intracellular caspase signaling to promote apoptosis [108]. In fact, infarcted cardiomyocytes can send exosomes to nearby cardiomyocytes, promoting apoptosis. Similarly, exosomes from infarcted cardiomyocytes contain abundant miR-19a-3p, which downregulates HIF-1α to inhibit proliferation of ECs and angiogenesis [109].

The same hypoxia that triggers cardiomyocyte apoptosis after MI upregulates the lncRNA AK139128 in cardiomyocytes and increases its levels in exosomes secreted by these cells. This lncRNA promotes apoptosis and inhibits proliferation of CFs, exacerbating myocardial injury after infarction [110]. However, hypoxia also activates transforming growth factor β1 (TGF-β1) to inhibit the apoptosis of CFs [132]. Interestingly, TGF-β signaling can interact with the Wnt signaling pathway and plays a key role in the differentiation of CFs [133]. This highlights the complex interplay within the signaling pathway network, which should be thoroughly explored in future studies.

## Regulation of inflammation

AMI temporarily increases the production of exosomes and microvesicles, mainly by cardiomyocytes and ECs. These vesicles accumulate in the ischemic myocardium, where they are rapidly taken up by infiltrating monocytes, which release chemokines and pro-inflammatory cytokines [134]. Ferroptosis lowers the levels of miR-106b-3p in exosomes from cardiomyocytes, leading to activation of Wnt signaling and a shift of cardiac macrophages to the M1 phenotype, exacerbating myocardial inflammation [80].

On the other hand, exosomes from hypoxic cardiomyocytes promote macrophage polarization toward the M2 phenotype, attenuating hypoxia-induced cardiomyocyte injury [111]. Further research is needed to clarify the how these exosomes influence macrophage polarization.

## Other effects

Excessive activation of CFs leads to harmful cardiac remodeling that can culminate in cardiac fibrosis. Hypoxic cardiomyocytes secrete exosomes containing miR-208a, which are taken up by CFs, in which the miRNA promotes fibroblast proliferation and myofibroblast differentiation, exacerbating cardiac fibrosis and the associated deterioration of cardiac function [112].

On the other hand, exosomes from ischemic cardiomyocytes can protect myocardium from oxidation-induced injury; promote ECs proliferation, sprouting, and tube formation through circHIPK3; and stimulate neovascularization through miR-222 and miR-143 [113, 114]. These studies elucidate the intricacy of intercellular interactions among cardiac cells resulting from the overlapping stages of inflammation, fibrosis, and angiogenesis in damaged myocardium. They emphasize the significance of adopting a comprehensive approach to comprehend the multifaceted factors and mechanisms involved in MI.

Exosomes from cardiomyocytes can regulate cardiac cells but also MSCs. In co-cultures of ischemic cardiomyocytes with MSCs, the cardiomyocytes secreted exosomes containing the lncRNA KLF3-AS1, which upregulated signal transducer and activator of transcription 5B (STAT5B) in MSCs, leading them to secrete IGF-1 to promote repair of myocardial tissue damaged by I/R [115]. To explore therapeutic possibilities, future research should explore the full range of cells that exosomes from ischemic/hypoxic cardiomyocytes can target, as well as the resulting biological effects.

## Exosomes derived from cardiosphere-derived cells 

Cardiosphere-derived cells (CDCs) are a type of cardiac stromal/progenitor cells that exert beneficial immunomodulatory, anti-fibrotic and pro-regenerative properties in diseased cardiac and skeletal muscle [135]. Studies in various animal models of MI and phase I trials with patients have shown that CDCs can reduce scarring, prevent unfavorable remodeling and increase survival [136, 137]. As a result, phase II trials are underway to assess the ability of CDCs to reduce scarring after MI. It appears that exosomes secreted by CDCs explain most if not all the cardioprotective, anti-apoptotic and regenerative effects [138, 139].

## Inhibition of cardiomyocyte apoptosis

Exosomes secreted by CDCs under both hypoxic and normoxic conditions inhibit apoptosis in cardiomyocytes [116]. Exposing CDCs to hypoxia substantially increases levels of pro-angiogenic miRNAs such as miR-126, miR-130a, and miR-210 in their exosomes [117].

## Inhibit inflammation

After cardiac I/R, exosomes from CDCs localize to ischemic tissues, reduce infarct size, and polarize macrophages into a highly phagocytic state similar to M2. These macrophages express abundant anti-inflammatory genes, efficiently phagocytose necrotic cellular debris, and attenuate excessive inflammatory stress within the infarcted heart. These effects appear to be mediated at least in part by miR-181b within the exosomes; this miRNA downregulates protein kinase δ (PKCδ) in macrophages and polarizes macrophages toward a cardioprotective phenotype [119]. Besides, Exosome-enriched EVs derived from CDCs contain abundant non-coding Y RNA called EV-YF1, which upregulates the pro-inflammatory factor IL-10 in macrophages, helping protect cardiomyocytes from oxidative stress [118]. These results suggest the importance of exploring ncRNA cargo in exosomes more extensively.

## Other effects

The potent pro-angiogenic and anti-fibrotic properties of CDC-secreted exosomes can produce multiple beneficial effects, such as reducing scarring and improving contractile function when injected intramyocardially in an ischemic cardiomyopathy model [136]. This can be attributed to the fact that the new capillaries not only supply nutrients to the infarction border zone, but also provide energy for the differentiation of CFs, thereby compensating for the maintenance of cardiac structure and functional integrity [82, 140]. Additionally, through the reduction of myocardial fibrosis, they can enhance conduction and make the post-ischemic heart less susceptible to arrhythmias [141].

## Exosomes derived from ECs and endothelial progenitor cells

ECs regulate vascular tone, angiogenesis, homeostasis, and inflammation in vivo and are major players in cardiovascular physiology and pathophysiology. Given the ability of exosomes from ECs to protect the brain and kidney from I/R injury, several studies have explored their potential against myocardial I/R injury [142]. Endothelial progenitor cells (EPCs), which serve as precursors to ECs, also secrete exosomes that may have therapeutic benefits in treating injury following MI [143].

## Promotion of angiogenesis

ECs rely on exosomes, in part, to induce angiogenesis [144]. For example, the exosomal level of profilin 2 (PFN2) increased in the serum of post-MI patients and animal models during angiogenesis. When exosomes derived from endothelial cells overexpressing PNF2 were injected into MI mice, they delivered PNF2 to endothelial cells, promoting angiogenesis and protecting the cells from inflammatory damage through the PI3K-PFN2-extracellular regulated protein kinase (ERK) axis [120].

Although the functional substrates and potential molecular mechanisms of EPCs therapy for MI have not been fully elucidated, studies have shown that the angiogenesis effects of exosomes from EPCs are closely related to ECs. Exosomes from these progenitor cells contain miR-1246 and miR-1290, which upregulate E74-like ETS transcription factor 5 (ELF5) and specificity protein 1 (SP1), promoting the conversion of CFs to ECs, leading to angiogenesis [124]. The exosomes also contain miR-218-5p and miR-363-3p that upregulate p53 signaling and downregulate JMY to promote the mesenchymal-endothelial transition and angiogenesis [125].

## Inhibit cardiomyocyte death

Exosomes from ECs can inhibit apoptosis of cardiomyocytes, such as by stimulating PI3K/AKT signaling [121]. For example, exosomes from vascular ECs also contain the lncRNA LINC00174, which downregulates *P53* in cardiomyocytes, thereby inhibiting the AKT/ adenosine 5 ‘-monophosphate (AMP)-activated protein kinase (AMPK) pathway and attenuating cellular autophagy and apoptosis [122].

## Other effects

In the post-MI ischemic microenvironment, aggregation and hyperactivation of neutrophils will further exacerbate tissue damage. The number of circulating neutrophils is strongly correlated with the infarct size [145]. MI rapidly mobilizes neutrophils from the spleen into the peripheral blood, and one of the drivers of this process is vascular cell adhesion molecule-1 in exosomes secreted by injured ECs [123]. This study reinforces the idea that exosomes can strongly influence immune responses to MI.

## Exosomes derived from other cardiac cells

Other types of cardiac cells also secrete exosomes potentially useful for treating MI. For example, exosomes from cardiac resident progenitor cells contain pregnancy-associated plasma protein-A (PPAP-A) that promotes the release of IGF-1 and phosphorylation of AKT and ERK1/2, while reducing caspase activation, preventing cardiomyocyte apoptosis and enhancing ventricular function after I/R injury [126]. In fact, such exosomes can activate multiple signaling pathways to promote cardiomyocyte migration, survival and proliferation, as well as stimulate angiogenesis directly and indirectly through monocytes, thereby promoting tissue repair and regeneration in multiple ways [127].

Cardiac telocytes are cardiac interstitial cells in the cardiac mesenchyme that help guide myocardial development and that can promote myocardial regeneration after infarction. These cells secrete exosomes that can mitigate injury after MI, such as by carrying miRNA-21-5p, which downregulates cell death inducing protein 1 (*Cdip1*) gene to inhibit apoptosis of cardiac microvascular ECs and promote cardiac angiogenesis [128, 146].

Exosomes from cardiomyocytes can influence the proliferation and differentiation of CFs [112], and the converse is true: CFs can affect cardiomyocytes after I/R injury via a paracrine pathway [147], such as by delivering miR-133a, which downregulates ELAV-like RNA-binding protein 1 (ELAVL1), thereby inhibiting pyroptosis [129].

Although epicardial cells have rarely been studied as a source of exosomes, probably because these cells are largely quiescent in adults [148], exosomes from these cells can promote cardiomyocyte proliferation after myocardial injury [130]. These findings highlight the danger of neglecting “unremarkable” intracardial cell populations that may take on important therapeutic functions after MI. Future studies should explore the potential therapeutic usefulness of these exosomes.

## Application forms of exosomes in MI therapy

Data from several preclinical studies suggest that exosomes have advantages over cell-based therapies in terms of safety, low immunogenicity, and ease of storage, but there are still some inadequacies to be addressed [37]. Short half-life and low retention rate are among the drawbacks of direct exosome injection. Therefore, advanced engineering techniques are required to functionalize exosomes, enhancing their efficiency and potency as therapeutics for MI. Currently, the main forms of preclinical research applications of exosomes include using biocompatible and biodegradable delivery systems to help deliver exosomes or engineering exosomes to enhance their therapeutic capability.

## Exosome co-delivery system for application

The application of delivery systems can overcome the issue of exosomes' low tissue residence time and provide a controlled release system to maintain their biological activity, thereby improving therapeutic effects on the infarcted myocardium. The mechanical properties, biocompatibility, biostability, and unique maneuverability of biomaterials make them the optimal choice for delivery systems [149]. Delivery methods typified by hydrogels have demonstrated significant therapeutic potential in laboratory settings and preclinical studies [150–154]. Researchers have utilized biomaterials as a foundation to better address the complex physiological environment of the heart following MI by chemically modifying, connecting self-assembling peptides, adding therapeutic molecules, and other approaches. These strategies enhance the accumulation of exosome-based delivery systems at disease sites, ultimately improving therapeutic efficacy. We summarize the current delivery systems for MI therapy, focusing on MSC and cardiac cell-derived exosomes (Table 4). Notably, both Zhu and Cheng et al. demonstrated the safety, efficacy, and clinical feasibility of minimally invasive intrapericardial injections in a clinically relevant porcine model and further explored the potential for treatment in humans [155, 156].

**Table 4:** Different delivery systems for exosome treatment of MI animal models

Delivery system	System composition	Source of exosomes	Route of Administration	Animal model	Key features	Reference
Hydrogel delivery system	Peptide amphiphile/ growth hormone-releasing peptides/ NapFF peptide hydrogel	UMSCs	Intramyocardial injection	MI rat	This functional peptide hydrogel can significantly improve infarcted myocardial functionthan exosomes alone	[151]
	Aniline tetrame-epoxy macromer/thiolated hyaluronic acid/ CP05 peptide hydrogel	UMSCs	Intramyocardial injection	MI rat	The composite hydrogel-exosome system has good targeting and conductivity that matches native myocardium and can significantly improve cardiac function	[153]
	Angiogenin-1 hydrogel	Aslet-1 overexpression in MSCs	Intramyocardial injection	MI mouse	Angiogenin-1 hydrogel could notably retain exosomes at ischemic sites, which improved the survival and angiogenesis of ECs	[157]
	Methacrylic anhydride–hyaluronic acid hydrogel	MSCs	Intrapericardial injection	MI mouse, porcine	Intrapericardial injection allows the hydrogel to form a cardiac patch in the pericardial cavity	[155]
	Alginate hydrogel	ADMSCs	Intramyocardial injection	MI rat	Exosomes loaded with miRNA-126 and miRNA-146a mimics synergistically enhance cardiac regeneration and therapeutic effects, partly by upregulating PI3K/AKT signaling	[158]
	Hyaluronic acid hydrogel	MSCs	Intrapericardial injection	Heart failure rat, porcine	The hydrogel-exosome system reduces the size of the left ventricle, preserves ventricular wall thickness	[156]
	Shear-thinning gel	Allogeneic EPCs	Intramyocardial injection	MI rat	Allogeneic EPC-EVs elicit minimal immune activity and maintain therapeutic efficacy in post-MI model after at least 2 months of cryopreservation	[159]
Engineered cardiac scaffold	Decellularized cardiac tissue/ peptide hydrogel scaffold	ADMSCs	Placement of cardiac scaffold	MI porcine	This engineered cardiac scaffold ensures controlled local dosage and release of exosomes, generating a vascularised bioactive niche	[160]
Microneedle Patch	Biocompatible microneedle patch based on gelatin	UMSCs	Implantation of this patch in the infarcted region	MI mouse	Exosomes loaded with miR-29b mimics exert antifibrotic effects, partly through modulation of the TGF-β signaling pathway	[161]

*ADMSCs* adipose mesenchymal stem cells, *AKT* protein kinase B, *ECs* endothelial cells, *EPC* Endothelial progenitor cells, *EVs* extracellular vesicle, *MI* myocardial infarction, *MSCs* mesenchymal stem cells, *PI3K* phosphatidylinositol3-kinase, *TGF-β* transforming growth factor-beta, *UMSCs* umbilical cord mesenchymal stem cells

By combining with other therapeutic modalities, exosomes can be developed for a wider range of applications. Given their ability to regulate cellular function directly and effectively, exosomes-based delivery systems are currently commonly used in both drug carrier and therapeutic agents. As mentioned earlier, sequential administration of exosomes and BMSCs has shown improved recruitment and survival of BMSCs, providing a promising strategy for the co-delivery of exosomes and living cells in synergistic therapy for heart-related disease [75]. Similarly, Chachques et al. utilized the therapeutic properties of exosomes by seeding MSC-derived exosomes along with MSCs and macrophages into an elastomeric cardiowrap scaffold [162]. The results demonstrated that exosomes significantly enhanced the wound healing properties of MSCs and shifted macrophages towards an M2 phenotype. This suggests that exosomes enable the implementation of multi-mechanism therapeutic strategies by influencing multiple cellular functions, representing another promising direction for exosome-based delivery systems.

In future exosome research, there is a need for further exploration of the development of exosome-based delivery systems for disease therapy, the determination of optimal release periods for exosomes, and ways to improve loading efficiency, among other issues.

## Engineering exosomes for drug delivery

Exosome engineering technology not only enables exosomes to act as natural nanocarriers for delivering molecular drugs but also enhances the target specificity of exosomes through surface modifications, making them more clinically applicable.

Drug loading strategies can be divided into two categories: endogenous (collecting drug-laden exosomes after pretreatment of parental cells) and exogenous (directly loading drugs into isolated exosomes) [163]. The former allows exosomes to be loaded with drugs at the cellular level, resulting in stronger therapeutic effects [164–167]. However, exosomes obtained through pretreatment usually exhibit relatively low drug loading efficiency and may have uneven drug levels [163]. In contrast, exogenous strategies provide better control over drug loading and have also been proven effective in animal models [168, 169]. Various drug loading methods have been developed and are described in detail in the review by Xi et al. [170]. The effectiveness of exosomes as drug carriers in preclinical studies has been initially established, and further validation of long-term safety should be conducted in the future. To achieve maximum drug loading efficiency, it’s suggested to coat exosome to metal organic framework or inorganic nanoparticles, both of which possess high drug loading efficiency [171].

Surface modification can enhance the specific targeting property and residence time of exosomes in cardiac tissues, thus improving the issue of exosomes diffusing out of the infarcted heart following direct injection and avoiding off-target effects. For example, Lee et al. used iron oxide nanoparticles to pretreat BMSCs, followed by serial cell extrusion, resulting in exosome-mimetic nanovesicles with enhanced therapeutic molecules and higher targeting efficiency [172]. Magnetic guidance further increased their retention in the infarcted hearts of MI rats. Similarly, exosomes can be modified either through genetic engineering or direct modification to bind cardiac homing peptides on their surface, enabling them to specifically target and treat ischemic myocardium when administered via tail vein injection [173, 174]. Intravenous delivery is expected to avoid the trauma caused by myocardial injection and provides a new direction for exosome delivery. Additionally, surface modifications can ameliorate the issue of circulating clearance of exosomes by immune cells due to intravenous injection. Exosomes incorporating CD47 protein on the surface can be protected from phagocytosis of macrophages and monocytes and have a longer half-life in circulation [175]. These studies suggest that exosomes can be delivered in a more convenient, safe and feasible way, offering broader prospects for clinical applications of exosomes. From the current perspective, the development of engineering exosomes is still in its infancy, with high costs for translational research and clinical applications.

## Conclusions and outlook

The causes affecting the prognosis of MI are diverse, but a fundamental problem is the irreversible loss of cardiomyocytes and the formation of scar tissue. Also, dysregulation of immune pathways, impaired suppression of postinfarction inflammation, disturbed spatial inhibition of the inflammatory response, and overactive fibrosis can contribute to poor remodeling of the heart, which may eventually lead to serious consequences such as heart failure [62]. Stem cell therapy has opened up new perspectives for treating MI, but cells transplanted into the ischemic heart often show poor recruitment and survival [75]. A more effective alternative may be delivering the therapeutic components of exosomes secreted by these cells. This review highlights the diverse range of exosome sources and contents that can mitigate injury, promote tissue repair, and regenerate after MI.

Preclinical evidence of exosome efficacy in animal models of MI is abundant [104, 176], with MSC-derived exosomes showing strong potential in many studies [177]. Although most exosome research is preclinical, there have been clinical trials that have initially verified the safety of exosomes, proving that exosomes purified by current methods may already be safe to use in patients. For example, injecting exosomes from MSCs into the brain parenchyma of five patients with ischemic stroke did not lead to serious adverse effects within 3-month follow-up [178]. The only clinical trial using MSC-derived exosomes for MI treatment is still under recruitment (NCT05669144), and further longitudinal clinical studies are necessary to confirm the safety and efficacy of exosome therapy.

In the case of exosomes secreted by cardiac cells, their cargo can depend on the pathological state of the cells. This characteristic could potentially make them useful as biomarkers for timely diagnosis, staging, and monitoring of disease during treatment. For instance, multiple studies have shown that miR-208a in exosomes released from injured cardiomyocytes after MI has the advantages of strong myocardial specificity, high sensitivity and strong stability, and is positively correlated with the indexes of myocardial function before and after therapy [112, 179–181]. The functional diversity of exosomes secreted by cardiac cells also suggests that these various cell types should be further explored as potential sources of therapeutic exosomes.

Intensive research on the molecular content and biological function of exosomes has facilitated the development of various engineering approaches. These methods have enhanced the targeting or therapeutic benefits of exosomes, but most engineering methods are only suitable for small-scale laboratory use. To make exosomes suitable for clinical therapeutics, several challenges still need to be addressed. Firstly, current techniques to isolate exosomes are not compatible with large-scale production for the clinic. Appropriate production methods and quality standards need to be established for exosome purification and characterization to ensure efficacy, safety and cost-effectiveness. Secondly, the low yield of exosomes makes it challenging to achieve industrialization, which also puts forward higher requirements for their cellular source. The cellular sources of exosomes should undergo differentiation and division as little as possible, so genetic engineering should be explored to modify existing cell types, such as the recent immortalization of MSCs [182]. Another potential source of therapeutic exosomes is plants, as illustrated in the ability of exosome-like nanoparticles from garlic to reduce obesity in a mouse model [183]. Genetic engineering should also be explored for creating exosomes that target specific tissues within the target organ(s), reducing their accumulation in off-target tissues [174].

Exosomes derived from MSCs and cardiac cells appear poised to play an important role in treating MI and potentially other cardiovascular diseases in the future. The functional diversity of cardiac cells and the ability of their exosomes to either mitigate or exacerbate myocardial injury highlight the need for future research to investigate the specific effects of exosome cargo on particular signaling pathways. Additionally, future studies should explore methods for pretreating or genetically modifying parental cells in order to optimize the therapeutic effects of their exosomes.

## Data Availability

Not applicable.

## Abbreviations

ADAMTS16: A disintegrin and metalloproteinase with thrombospondin 16
ADMSCs: Adipose mesenchymal stem cells
AIFM3: Apoptosis-inducing factor, mitochondrion-associated 3
AKT: Protein kinase B
ALIX: Apoptosis-linked gene 2-interacting protein X
AMI: Acute myocardial infarction
AMPK: Adenosine 5 ‘-monophosphate (AMP)-activated protein kinase
Apaf1: Apoptotic protease activating factor- 1
ATF2: Activating transcription factor 2
Bak1: B-cell lymphoma-2 antagonist/killer 1
Bax: B-cell lymphoma-associated X
BMSCs: Bone marrow mesenchymal stem cells
CDCs: Cardiosphere-derived cells
Cdip1: Cell death inducing protein
CFs: Cardiac fibroblasts
CHK2: Checkpoint kinase 2
DMBT1: Deleted in malignant brain tumors 1
DMT1: Recombinant divalent metal transporter 1
Dyrk2: Dual specificity tyrosine (Y) phosphorylation regulated kinase 2
ECs: Endothelial cells
ELAVL1: ELAV-like RNA-binding protein 1
ELF5: E74-like factor 5
EPCs: Endothelial progenitor cells
ERK: Extracellular regulated protein kinase
EVs: Extracellular vesicles
EZH2: Enhancer of zeste 2 polycomb repressive complex 2
Fasl: Factor related apoptosis ligand
FNDC5: Fibronectin type III domain-containing protein 5
FOXO1: Forkhead box O1
FOXO3: Forkhead box O3
GSDMD: Gasdermin D
GSK3 β: Glycogen synthase kinase 3β
HIF-1 α: Hypoxia-inducible factor 1
HMGA2: High mobility group AT-hook 2
HO-1: Heme oxygenase 1
I/R: Ischemia/Reperfusion
IGF-1: Insulin-like growth factor- 1
IL: Interleukin
JMY: Regulatory protein
JNK: C-Jun N-terminal kinase
MAP3K2: Mitogen-activated protein kinase kinase kinase 2
MAPK: Mitogen-activated protein kinase
MEKK1: Mitogen-activated protein kinase kinase kinase 1
MI: Myocardial infarction
miRNA: Micro RNA
MKK4: Mitogen-activated protein kinase kinase 4
MSCs: Mesenchymal stem cells
MVB: Multivesicular body
ncRNA: Non-coding RNA
NF-κB: Nuclear factor-κB
NLRP3: NOD-like receptor thermal protein domain associated protein 3
Nrf2: Nuclear factor erythroid 2-related factor 2
p-AKT: Phosphorylated protein kinase B
PAPP-A: Pregnancy-associated plasma protein-A
Pdcd4: Programmed cell death 4
PDGFR- β: Platelet-derived growth factor receptor-β
PFN2: Profilin 2
PI3K: Phosphatidylinositol3-kinase
PKC δ: Protein Kinase C δ
PP2A: Protein phospholipase 2A
Pten: Phosphatase and tensin homolog
RAS: Renin-angiotensin system
ROS: Reactive oxygen species
S1P: Sphingosine 1-phosphate
S1PR1: Sphingosine-1-phosphate receptor 1
Sirt1: Sirtuin 1
SK1: Sphingosine kinase 1
Smad: Mothers against decapentaplegic homolog
SOCS2: Suppressor of cytokine signaling 2
SOX6: SRY (sex determining region Y)-box 6
SP1: Specificity protein 1
SRSF1: Serine and arginine rich splicing factor 1
STAT5B: Signal transducer and activator of transcription 5B
TGFBR2: Transforming growth factor-beta receptor type 2
TGF-β1: Transforming growth factor-beta 1
TIMP2: Tissue inhibitor of metalloproteinase 2
TLR4: Toll-like receptor 4
TNF: Tumor necrosis factor
TSG101: Tumor susceptibility gene 101
UMSCs: Umbilical cord mesenchymal stem cells
VCAM-1: Vascular cell adhesion molecule 1
VEGF: Vascular endothlial growth factor
VEGFA: Vascular endothlial growth factor A
XIAP: X-linked inhibitor of apoptosis protein
